# Is team-based learning an alternative approach for UK undergraduate dental education? A scoping review of the literature

**DOI:** 10.1038/s41415-023-6615-x

**Published:** 2024-01-12

**Authors:** Benjamin James Trill, Bal Panesar, Manas Dave, Reza Vahid Roudsari, Hanieh Javidi

**Affiliations:** 41415462852001 Clinical Lecturer in Adult Oral Health, Division of Dentistry, School of Medical Sciences, University of Manchester, Manchester, M15 6FH, UK; 41415462852002 Clinical Lecturer, Division of Dentistry, School of Medical Sciences, University of Manchester, Manchester, M15 6FH, UK; 41415462852003 Lecturer in Dental Education and Speciality Registrar in Oral and Maxillofacial Pathology, Division of Dentistry, School of Medical Sciences, University of Manchester, Manchester, M15 6FH, UK; 41415462852004 Professor of Dental Education and Honorary Consultant in Restorative Dentistry, Division of Dentistry, School of Medical Sciences, University of Manchester, Manchester, M15 6FH, UK; 41415462852005 Senior Clinical Lecturer and Honorary Consultant in Orthodontics, Division of Dentistry, School of Medical Sciences, University of Manchester, Manchester, M15 6FH, UK

## Abstract

**Introduction** Team-based learning (TBL) is a dialectic, student-focused method of teaching which has become increasingly popular in international institutions for delivering undergraduate dental education. Despite several dental schools in the UK using dialectic teaching methods, such as problem-based learning, none appear to use TBL.

**Aims** This scoping review aims to identify the literature investigating the use of TBL compared with other teaching pedagogies in delivering undergraduate dental education.

**Methods** The Preferred Reporting Items for Systematic Reviews and Meta-Analyses Extension for Scoping Reviews guidelines were adopted. A search strategy was developed using appropriate MeSH (medical subject headings) terms and key words. Medline, Scopus and the Cochrane Databases were searched.

**Results** Overall, five studies were identified for inclusion. Of these, three studies compared TBL to traditional, didactic teaching methods (such as lectures) and found both student satisfaction and student performance to be greater with TBL. The remaining two studies compared TBL to other dialectic methods of teaching. The results on student performance in these studies were conflicting.

**Conclusions** There is some limited but promising evidence that TBL is effective at delivering undergraduate dental education; however, the scarcity of research evidence highlights the need for more robust exploration.

## Key points

- There is some evidence that team-based learning used to deliver undergraduate dental education leads to improved student satisfaction and student performance compared to traditional, didactic methods of teaching.
- There is limited research evidence comparing team-based learning to other dialectic methods of teaching in undergraduate dentistry.
- More robust research is required to explore the effectiveness of team-based learning in undergraduate dental education compared to other dialectic methods of teaching.

## Introduction

Traditionally, healthcare pedagogy has been delivered using didactic methods of teaching, centred on the transfer of information from teachers to students (usually in the form of lectures). However, for several decades now, there have been strong calls from both the medical and dental world encouraging transformation and placing the onus of learning on students themselves.^1^^,^^2^ Our healthcare environments today require professionals that are not only competent but are also able to work effectively in interprofessional teams to help deliver high-quality, cost-effective patient care. The widespread availability and use of the internet and digital platforms has revolutionised the progression and dissemination of medical and dental research findings, and our healthcare professionals must now be equipped with the ability to problem solve, critically appraise information, and where possible, ensure they are providing care which is supported by research evidence.^1^^,^^3^ To this effect, we have witnessed a paradigm shift in dental education methods, with learner-centred models, such as problem-based learning (PBL), enquiry-based learning (EBL) and case-based learning (CBL), being introduced.^4^^,^^5^^,^^6^^,^^7^^,^^8^^,^^9^An overview of these three pedagogies is outlined in Table 1. While there are numerous similarities between these three pedagogies, there are also subtle differences.^10^^,^^11^^,^^12^^,^^13^^,^^14^ Within the UK, several universities have deployed PBL and EBL teaching methods to aid the delivery of their undergraduate dental curriculums.^6^^,^^15^^,^^16^^,^^17^ These dialectic methods are fundamentally based on the use of small groups (typically 5-8 students together with a facilitator), to address theoretical and clinical topics, generally supported by appropriately selected clinical cases. Students approach these through a combination of enquiry, independent study, discussion and debate.^17^ The overall aim is a far more interactive and dynamic form of learning; when compared to lecture-based teaching, PBL, EBL and CBL are active rather than passive educational approaches to teaching. However, despite the perceived advantages of these methods,^18^^,^^19^ they require a relatively high number of facilitators and a large number of small rooms to accommodate groups, which in itself can present a logistical challenge to educational institutions.^19^ Furthermore, there can be issues with educational quality standardisation, as there may be variation in the ability of small groups to interpret clinical cases and adequately identify the intended learning objectives.^19^^,^^20^ Scenarios can arise where groups learn vastly different content, especially if the facilitator is not adequately trained to guide discussions.^19^^,^^20^ To address some of these drawbacks, attention has now been drawn to an alternative learner-centred teaching model known as team-based learning (TBL).^21^^,^^22^

**Table 1:** Definitions of constructivist philosophy teaching approaches including EBL/IBL, PBL and CBL

Enquiry-based learning (UK) Inquiry-based learning (US)	This is a student-centred active learning approach where students ask a question or series of questions on a topic and/or scenario before research and investigations of the topic and/or scenario. This results in the generation of new knowledge. The tutor provides information and supports the process, ensuring clarity of thinking^12^^,^^14^
Problem-based learning	A student-centred active learning approach where the student conducts research and develops skills and knowledge to find a solution to a defined, often-real world problem. This results in the development of a knowledge base. In contrast to EBL, the tutor should not provide information about the problem but supports the process and ensures clarity of thinking^12^ Some authors highlight that PBL is a form of EBL as it is enquiry-focused learning^10^^,^^13^^,^^14^
Case-based learning	A student-centred active learning approach where the student is presented with a ‘real-life' case that stimulates exploration of a desired topic(s), developing knowledge and promoting the application of current knowledge^11^^,^^12^ Some authors highlight that CBL is a form of EBL as it is enquiry-focused learning^14^

**Fig. 1:**
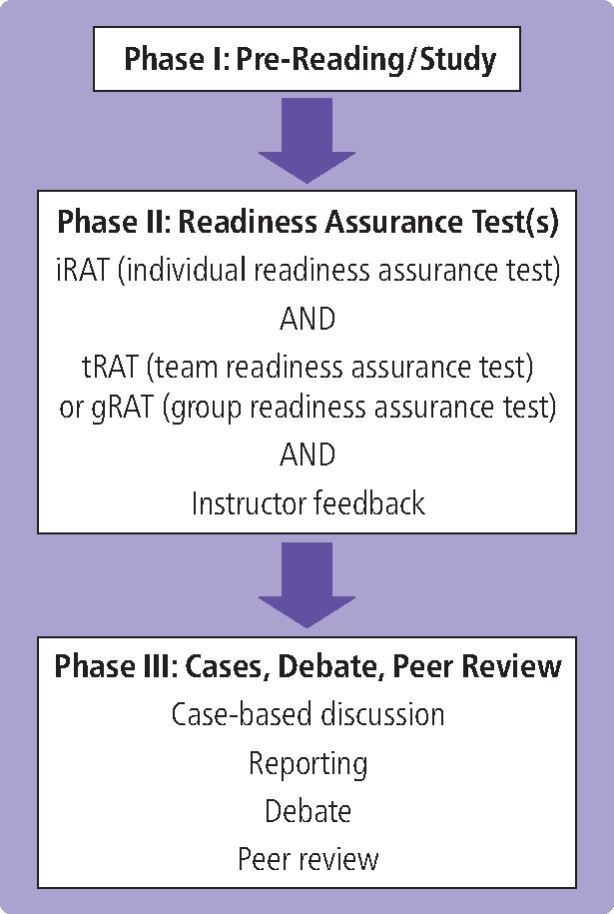
A flowchart to demonstrate the three phases of TBL and the processes involved in each phase

TBL involves large group classes (which can be several hundred students) divided into smaller teams (typically 5-7), together with a smaller number of facilitators (typically 2-3).^21^^,^^22^ Prior to the session, students are expected to complete pre-reading in preparation for an assessment known as the individual readiness assurance test (iRAT).^21^^,^^22^ This is often in the form of a multiple-choice assessment and is used to evaluate concepts learnt in the pre-reading material. Following completion of the iRAT, the students convene in their allocated teams and answer the questions together in a team/group readiness assurance test (tRAT/gRAT).^21^^,^^22^ Subsequently, the teams are presented with real-life problem scenarios with multiple solutions, which they are able to discuss, rationalise and problem-solve together before the teams are able to debate the solutions that they have synthesised. The final step in the TBL process is peer review, whereby students provide feedback to each other, including their ability to work effectively in a team.^21^^,^^22^ The process of TBL is summarised in Figure 1.

In recent years, TBL has gained popularity in medical education, and there is now evidence emerging that students favour this over methods such as PBL.^23^ There are studies which have found that medical students prefer the structure and format of TBL sessions and feel these to be more conducive to learning, engagement and participation when compared to PBL.^23^^,^^24^ A recent systematic review exploring the impact of TBL among healthcare professionals found evidence that academic performance was significantly improved among TBL groups when compared to lecture-based teaching groups.^25^ While the evidence to date supporting the use of TBL in healthcare education is promising, much of this research has been based on the use of TBL in the development of medical, nursing and allied healthcare professionals.^25^^,^^26^^,^^27^As such, there is a real need to determine the effectiveness of TBL in delivering undergraduate dental education. The aim of this scoping review is to:

- Evaluate the current research exploring the effectiveness of TBL versus other teaching pedagogies in delivering undergraduate dental education
- Identify the strengths and weaknesses within the current evidence base
- Stimulate discussion on the suitability of TBL in delivering undergraduate dental education within the UK.

## Methods

### Information sources and search

Scoping reviews provide an ideal tool for determining the nature of literature available in a particular subject area.^28^ As such, this was the most appropriate research methodology for addressing the objectives of this study. This scoping review was conducted using the Preferred Reporting Items for Systematic Reviews and Meta-Analyses (PRISMA) Extension for Scoping Reviews guidelines.^29^ The following electronic databases were searched: Medline, Cochrane and Scopus (May 2023). The review was limited to literature published from inception through to 2 May 2023. Two reviewers (BJT and HJ) searched the databases using the MeSH (medical subject headings) terms and key words ‘dentistry' OR ‘dental' AND ‘team-based learning' OR ‘TBL' OR ‘iRAT' OR ‘tRAT'.

### Inclusion criteria

- Articles written in the English language
- Dentistry at undergraduate level (any year of study)
- Studies comparing TBL with any other form of teaching pedagogy.

### Exclusion criteria

- Articles written in any language other than English
- Studies focusing on postgraduate courses
- Studies where TBL was not compared to another form of teaching pedagogy.

### Screening and data collection

Assessment and screening of studies was completed independently by three authors (BJT, BP and HJ).^30^ A selection protocol based on the inclusion/exclusion criteria of the review was designed and piloted on the first 50 studies returned from the electronic database search. This was designed and used to provide a consistent method for selecting studies that appropriately addressed the objectives of this study. The selected articles had their full text read by two authors (BJT and HJ) and were assessed for final inclusion. Any discrepancies were planned for resolution through discussion and involvement of a third author (RVR). The reference lists of all included articles were researched for any additional relevant articles.

A data collection sheet was developed using the guidelines described by Free *et. al.*^31^ and piloted before implementation. The characteristics and findings of all the studies included in the review were extracted using the data collection sheet. This was conducted independently and in duplicate by two authors (BJT and HJ). Ethical approval was not required because this study retrieved and synthesised data from previously published studies.

## Results

### Selection and characteristics of source of evidence

**Fig. 2:**
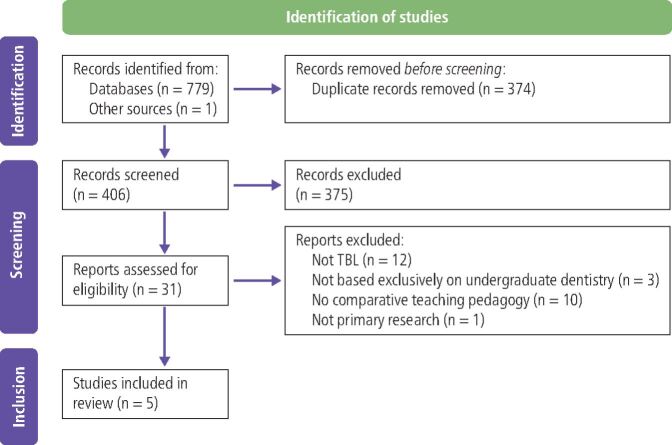
PRISMA flow diagram presenting the selection process for this scoping review

The electronic database searches yielded a total of 780 articles. Following removal of duplicates, 406 articles were returned. Following initial title and abstract screening, 375 articles were removed. A total of 31 full-text articles were reviewed with the eligibility criteria and 26 excluded. In total, five studies were included in this scoping review.^32^^,^^33^^,^^34^^,^^35^^,^^36^ The PRISMA diagram in Figure 2 provides a summary of the selection process.

The included studies are summarised in Table 2. They were conducted in the USA^32^^,^^33^ (n = 2), Japan^35^^,^^36^ (n = 2) and Saudi Arabia (n = 1).^34^ The effectiveness of TBL in teaching prosthodontics was assessed in three studies,^32^^,^^35^^,^^36^ while one study focused on the teaching of oral and systemic topics.^33^ In one of the studies, it was unclear which discipline of dentistry was being taught.^34^ Of the selected studies, three compared TBL to traditional methods (lectures),^32^^,^^34^^,^^36^ one compared TBL to case-based learning,^33^ and one compared TBL to flipped classroom (flipped classroom is a blended approach where the student watches a series of videos or digital media before attending a class or workshop).^35^^,^^37^

**Table 2:** Characteristics of selected studies included in the scoping review

Study (country)	Dental speciality/ discipline	Comparison group	Number of participants recruited	Outcomes	Results	Additional findings
Echeto *et al.*, 2015^32^ (USA)	Prosthodontics (removable partial dentures)	Conventional: lectures, quizzes and laboratory sessions	TBL: n = 82 (response: 78/82) Conventional: n = 84 (response: 79/84)	Knowledge retention: examination results	A significantly greater (p = 0.002) number of students who received TBL teaching passed the examination vs conventional teaching The mean score for the TBL class was statistically significantly higher than the conventional class (p <0.001) Odds ratio 2.75 times more likely to fail if conventional teaching approaches had been taken vs TBL	
Haley *et al.*, 2020^33^ (USA)	Oral and systemic topics in dentistry	CBL Two semester long courses - one CBL and one TBL	Original enrolment: n = 68 67 students score comparison 66% (44/67) completed both surveys and were included in analysis CBL faculty: n = 16 (response: 14/16) TBL faculty: n = 6 (response: 5/6)	Student satisfaction (through survey) Examination performance Faculty perceptions (through survey)	Student satisfaction: higher in CBL vs TBL (Mann-Whitney U test = 882.0; p <0.001) CBL classes contributed more to their learning (U = 746.0; p <0.001), they understood concepts better in CBL (U = 899.0; p <0.001) and had higher level of improvement in communication abilities with CBL (U = 891.0; p <0.001) Most students preferred the small group teaching of CBL and there was a lot of dissatisfaction with the perceived rigidity of TBL Overall performance was better in TBL (mean grade 86%) vs CBL (mean grade 83%) (p = 0.046)	Fewer faculty required for TBL More preparation time was required for faculty in TBL vs CBL (p = 0.03)
Nawabi *et al.*, 2021^34^ (Saudi Arabia)	Unclear	Traditional lecture-based	n = 147 (response: 120/147)	Student perceptions Examination performance	Student perception of TBL superior to lectures Significantly (p <0.05) higher grades in TBL examinations vs lecture-based	Women obtained significantly higher grades in TBL (p <0.05)
Nishigawa *et al.*, 2017^35^ (Japan)	Prosthodontics (fixed)	Flipped classroom (e-learning, individual testing, feedback, explanation and individual instruction)	n = 41	Examination performance	No statistically significant difference between examination performance (p = 0.848)	
Takeuchi *et al.*, 2015^36^ (Japan)	Prosthodontics (fixed)	Traditional lecture-based Three traditional lecture classes followed by seven TBL classes (one class for explaining TBL format)	n = 36 (response: 36/36)	Student perceptions Examination scores	Students perceived a higher active attitude in a class and felt more prepared (p <0.01) with TBL compared to traditional style teaching Degree of achievement from the class was higher in TBL (p <0.05) Examination scores were higher in TBL compared to traditional teaching (p <0.01)	

Although the studies used different outcomes to assess the effectiveness of TBL versus other teaching pedagogies, these outcomes were largely found to fall into two main themes, namely, student satisfaction/perception and examination performance. In four of the studies, examination performance was found to be significantly better in favour of TBL,^32^^,^^33^^,^^34^^,^^36^ while one study found no statistically significant difference between material taught using TBL versus the flipped classroom method.^35^

Student perception of TBL was assessed in two of the studies,^34^^,^^36^ both of which found TBL to be perceived more favourably than traditional, lecture-based approaches. Student satisfaction was assessed in a single study which compared TBL with CBL and found student satisfaction to be higher in CBL.^33^

## Discussion

To our knowledge, this is the first scoping review of the literature that has investigated the use of TBL in undergraduate dental education. TBL has gained popularity as a teaching method in allied health professions^25^ and this review has found some evidence that TBL is gradually being used as a teaching pedagogy in undergraduate dental education. While five studies were identified for inclusion,^32^^,^^33^^,^^34^^,^^35^^,^^36^ there was substantial heterogeneity in both study design and data analysis, which makes direct comparison of the data from each of these studies difficult. Furthermore, the sample size of most of these studies was relatively small and the teaching was limited to isolated, short dental courses, which limits extrapolation of these findings to a full undergraduate dental curriculum.

The results of the studies included in this review suggest that TBL in undergraduate dental education leads to improved student performance when compared to traditional, lecture-based approaches,^32^^,^^34^^,^^36^ but there is limited evidence comparing the effectiveness of TBL versus other constructivist-focused pedagogy, such as CBL, PBL or EBL. There were two studies which compared active constructivist approaches, specifically TBL with CBL,^33^ and TBL with flipped classroom.^35^ The results of these studies in relation to student performance were conflicting. Where TBL was compared to CBL, the authors reported a statistically significant (p = 0.046) improvement in student performance with TBL.^33^ However, on close examination, it is clear that this difference is minimal (86% vs 83%) and therefore unlikely to be consequential.^33^ Where TBL was compared to the flipped classroom approach, there was no significant difference found in examination performance (p = 0.848).^35^ As such, based on the current evidence to date, we cannot draw any robust conclusions regarding the impact of TBL on student performance when compared to other constructivist teaching pedagogies in undergraduate dental education.

Based on the studies identified for inclusion in the review, it appears that student satisfaction was perceived to be higher for TBL when compared to traditional lecture-based approaches^34^^,^^36^ but inferior when compared to another constructivist learning approach (CBL).^33^ The single study which compared two constructivist approaches (TBL vs CBL) highlighted a number of issues with TBL, notably a dissatisfaction in TBL rigidity and a preference for smaller group sizes in CBL.^33^ Overall, there appears to be limited evidence comparing student perceptions/satisfaction of TBL with other teaching pedagogies in undergraduate dental education. That said, however, there are studies which have looked at student satisfaction with TBL in undergraduate dental education and found high levels of satisfaction, but such studies have assessed TBL alone and have failed to compare this to other teaching pedagogies.^38^^,^^39^

In the UK, dentistry is a healthcare modality that has traditionally been provided within the NHS. Over the last 12 months, it has become increasingly publicised that NHS dentistry is in a precarious state, and while several potential solutions have been put forward and discussed on public, professional and political platforms, one potential solution has been to increase the number of undergraduate dental places within our educational institutions.^40^ While such a change may increase the size of our future dental workforce, it is paramount that institutions are able to accommodate these increasing numbers while still providing high-quality training and ensuring that these increased numbers do not adversely affect the competency and proficiency of graduating dental students. It is therefore pertinent, now more than ever, that we ensure the teaching pedagogies used to deliver undergraduate dental education are effective and make efficient and appropriate use of the resources available within our educational institutions. At present, multiple institutions in the UK utilise constructivist teaching approaches such as PBL and EBL in undergraduate dental programmes,^15^^,^^17^^,^^41^ and while these are generally considered more favourable than traditional, lecture-based methods, they are fraught with their own distinct and logistical challenges.^19^^,^^42^^,^^43^ These range from lack of educational standardisation to the increasing number of rooms needed to accommodate the small group discussions and the large number of facilitators required. One could argue that in contrast to this, TBL is a teaching pedagogy that maintains the benefits of a constructivist approach to teaching while requiring only a minimal number of expert facilitators. It has the added benefit that every student receives the same level of expert input, and as such, institutions can potentially be more confident about the standardisation of teaching, leading to improved levels of quality assurance.

## Conclusion

As outlined above, TBL may have the potential to provide a more effective constructivist teaching methodology for delivering undergraduate dental education. Based on the literature identified in this scoping review, it appears that there is some evidence that TBL may be effective in delivering undergraduate dental education, but this evidence is limited and fraught with several limitations. Overall, most of the studies identified had poor research methodology (there was a lack of randomised controlled trials), were based on small sizes and had delivered TBL to very small portions of the dental curriculum. In addition to this, in most of the studies, the outcomes were focused on a combination of student perception, satisfaction and examination performance, and no regard was given to the perception of educators/institutions. While not entirely conclusive, there is sufficient evidence to support further assessment of TBL in delivering undergraduate dental education using appropriate research methodology and utilising outcomes relevant to all stakeholders.
